# Supplementary material for: Multiplexed representation of others in the hippocampal CA1 subfield of female mice
Source: Nat Commun. 2024 May 2;15:3702. doi: 10.1038/s41467-024-47453-8 (PMC11065873; doi:10.1038/s41467-024-47453-8)
Supplement: Supplementary file 1 — Supplementary Information [file 41467_2024_47453_MOESM1_ESM.pdf]

Supplementary Information

Multiplexed representation of others in the hippocampal CA1 subfield of female mice

Xiang Zhang[1, 2], Qichen Cao[1, 2, 3], Kai Gao[1, 2, 3], Cong Chen[1, 2], Sihui Cheng[1, 2, 3], Ang Li[1, 2, 3], Yuqian Zhou[1, 2], Ruojin Liu[1, 2, 3], Jun Hao[1, 2, 3], Emilio Kropff[4*], Chenglin Miao[1, 2, 3, 5*]

1. State key laboratory of Membrane biology, School of Life Sciences, Peking university, Beijing, China
2. PKU-IDG/McGovern Institute for Brain Research, Peking university, Beijing, China
3. Peking-Tsinghua Center for Life Sciences, Beijing, China
4. Leloir Institute/IIBBA-CONICET, Buenos Aires, Argentina
5. Chinese Institute for Brain Research (CIBR), Beijing, China

- Supplementary Figures 1-14
- Supplementary Movies 1-2

Supplementary Figures

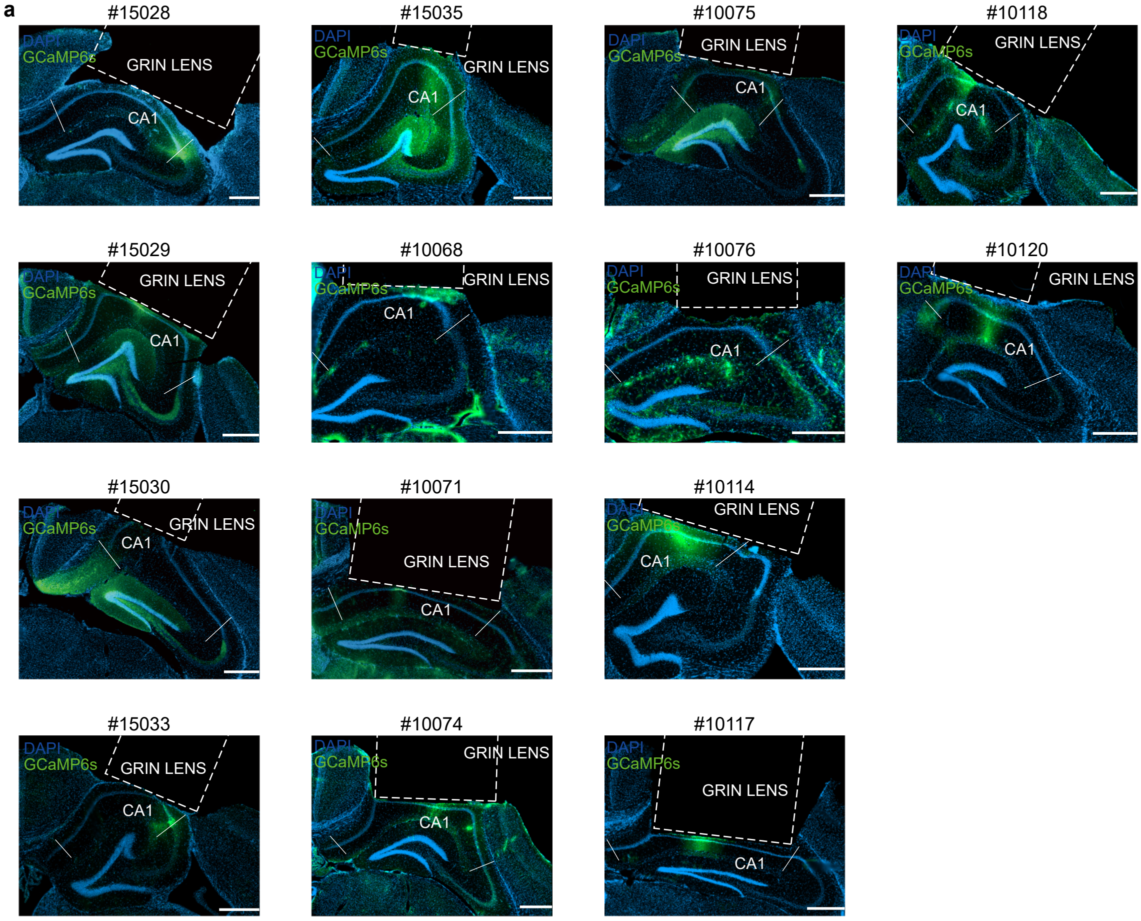

Supplementary Figure 1. Histological reconstructions of CA1-targeted imaging.

a, Representative histological slices showing GCaMP6s infected cells (green) in the CA1 region. Dashed lines indicate the position of the GRIN lens. Scale bar: 500 μm. Note that all GRIN lenses are 1.8 mm in diameter and that the different size in lens trace is due to the fact that not all lenses were perfectly centered in the region of interest.

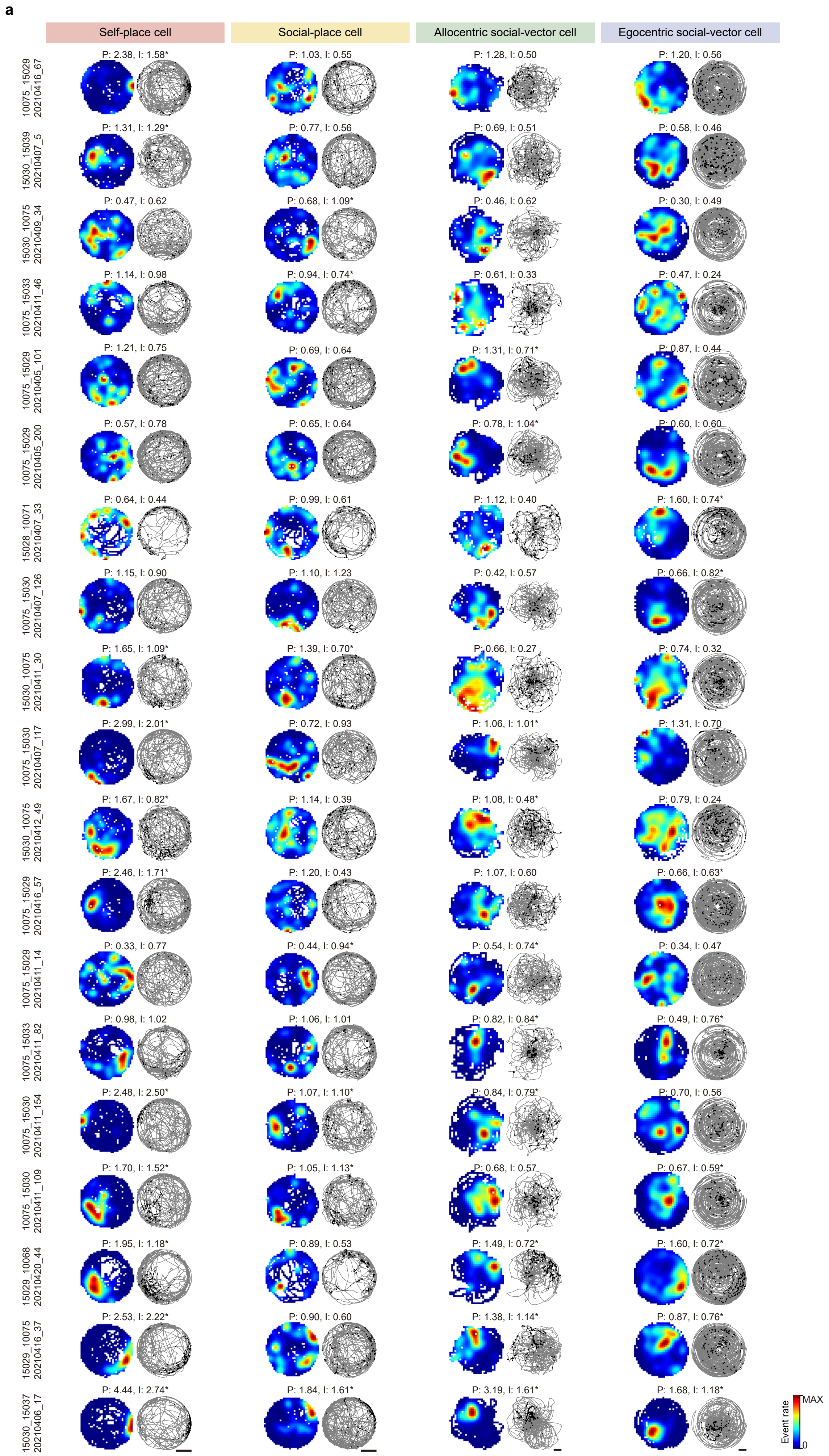
a
Self-place cell
Social-place cell
Allocentric social-vector cell
Egocentric social-vector cell
10075_15029 20210416_67
P: 2.38, I: 1.58*
P: 1.03, I: 0.55
P: 1.28, I: 0.50
P: 1.20, I: 0.56
15030_15039 20210407_5
P: 1.31, I: 1.29*
P: 0.77, I: 0.56
P: 0.69, I: 0.51
P: 0.58, I: 0.46
15030_10075 20210409_34
P: 0.47, I: 0.62
P: 0.68, I: 1.09*
P: 0.46, I: 0.62
P: 0.30, I: 0.49
10075_15033 20210411_46
P: 1.14, I: 0.98
P: 0.94, I: 0.74*
P: 0.61, I: 0.33
P: 0.47, I: 0.24
10075_15029 20210405_101
P: 1.21, I: 0.75
P: 0.69, I: 0.64
P: 1.31, I: 0.71*
P: 0.87, I: 0.44
10075_15029 20210405_200
P: 0.57, I: 0.78
P: 0.65, I: 0.64
P: 0.78, I: 1.04*
P: 0.60, I: 0.60
15028_10071 20210407_33
P: 0.64, I: 0.44
P: 0.99, I: 0.61
P: 1.12, I: 0.40
P: 1.60, I: 0.74*
10075_15030 20210407_126
P: 1.15, I: 0.90
P: 1.10, I: 1.23
P: 0.42, I: 0.57
P: 0.66, I: 0.82*
15030_10075 20210411_30
P: 1.65, I: 1.09*
P: 1.39, I: 0.70*
P: 0.66, I: 0.27
P: 0.74, I: 0.32
10075_15030 20210407_117
P: 2.99, I: 2.01*
P: 0.72, I: 0.93
P: 1.06, I: 1.01*
P: 1.31, I: 0.70
15030_10075 20210412_49
P: 1.67, I: 0.82*
P: 1.14, I: 0.39
P: 1.08, I: 0.48*
P: 0.79, I: 0.24
10075_15029 20210416_57
P: 2.46, I: 1.71*
P: 1.20, I: 0.43
P: 1.07, I: 0.60
P: 0.66, I: 0.63*
10075_15029 20210411_14
P: 0.33, I: 0.77
P: 0.44, I: 0.94*
P: 0.54, I: 0.74*
P: 0.34, I: 0.47
10075_15033 20210411_82
P: 0.98, I: 1.02
P: 1.06, I: 1.01
P: 0.82, I: 0.84*
P: 0.49, I: 0.76*
10075_15030 20210411_154
P: 2.48, I: 2.50*
P: 1.07, I: 1.10*
P: 0.84, I: 0.79*
P: 0.70, I: 0.56
10075_15030 20210411_109
P: 1.70, I: 1.52*
P: 1.05, I: 1.13*
P: 0.68, I: 0.57
P: 0.67, I: 0.59*
15029_10068 20210420_44
P: 1.95, I: 1.18*
P: 0.89, I: 0.53
P: 1.49, I: 0.72*
P: 1.60, I: 0.72*
15029_10075 20210416_37
P: 2.53, I: 2.22*
P: 0.90, I: 0.60
P: 1.38, I: 1.14*
P: 0.87, I: 0.76*
15030_15037 20210406_17
P: 4.44, I: 2.74*
P: 1.84, I: 1.61*
P: 3.19, I: 1.61*
P: 1.68, I: 1.18*
Event rate
MAX
0

Supplementary Figure 2. Maps in multiple reference frameworks.

a, Representative examples (one cell per row) of rate maps and self or social trajectory in each of the four reference frameworks (columns). Left subpanel: rate map. Right subpanel: trajectory in the corresponding reference framework (grey) and positions where calcium transients were observed (black dots). Peak rate (P) and information content (I) are indicated for each map, and an asterisk indicates that information content is significantly higher than the shuffled distribution. Scale bar: 20cm.

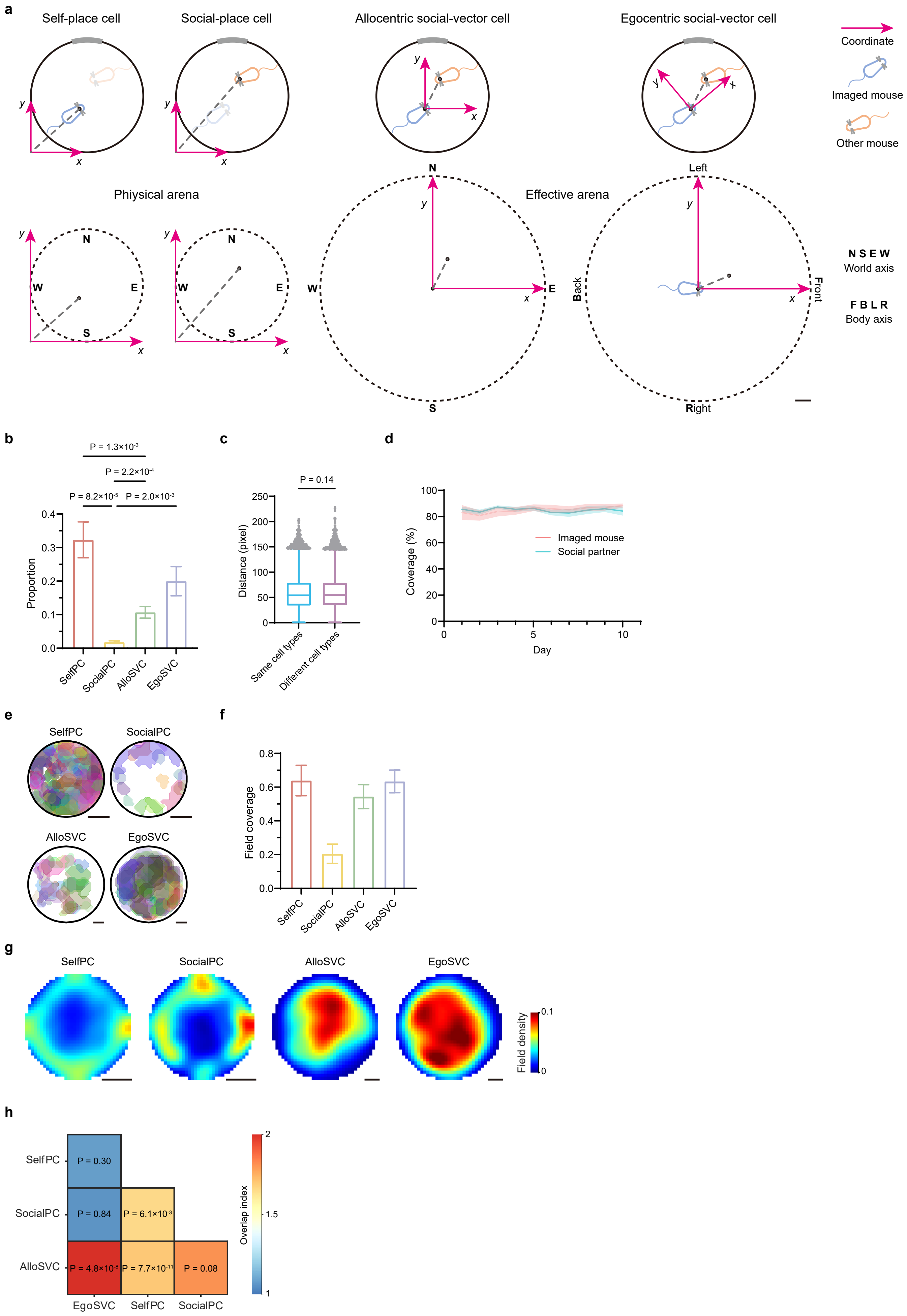
a
Self-place cell
Social-place cell
Allocentric social-vector cell
Egocentric social-vector cell
Coordinate
Imaged mouse
Other mouse
Phiysical arena
Effective arena
N S E W
World axis
F B L R
Body axis
N
S
W
E
Left
Right
Back
Front
b
P = 1.3×10-3
P = 2.2×10-4
P = 8.2×10-5
P = 2.0×10-3
Proportion
SelfPC
SocialPC
AlloSVC
EgoSVC
c
P = 0.14
Distance (pixel)
Same cell types
Different cell types
d
Coverage (%)
Imaged mouse
Social partner
Day
e
SelfPC
SocialPC
AlloSVC
EgoSVC
f
Field coverage
g
SelfPC
SocialPC
AlloSVC
EgoSVC
Field density
h
SelfPC
SocialPC
AlloSVC
EgoSVC
P = 0.30
P = 0.84
P = 6.1×10-3
P = 4.8×10-8
P = 7.7×10-11
P = 0.08
Overlap index

Supplementary Figure 3. Place cells collected during social interaction.

a, Scheme of the positions of an imaged mouse and its conspecific (top) and the corresponding extension of spatial maps (bottom) in each coordinate system (columns). Note that maps for selfPC and socialPC can be thought of as spanning the physical arena, while maps for alloSVC and egoSVC, which code for the position of one mouse relative to the other, need to be considered in an 'effective arena' with a four times larger area. Scale bar: 10 cm. **b**, Fraction of imaged cells falling into each category (similar to Fig. 1**g**) but grouping data by mouse (mean ± s.e.m.; n = 18 imaged mice. RM one-way ANOVA, df = 3, F (1.5, 26.2) = 14.04, p = 2.0×10^{-4}. Holm-Šídák's multiple comparisons test for each cell type, p value indicated). **c**, Distance in pixels between pairs of centroids of somas belonging to the same (blue) or to a different (violet) category (median ± i.q.r., Mann Whitney test, two tailed, Mann-Whitney U = 3252835237, Cliff's Delta = -4.4×10^{-3}, p = 0.14). **d**, Percentage of the physical arena covered by imaged mice (red) and social partners (blue) during the free social interaction paradigm (mean ± s.e.m.; 10 imaged mice and 10 social partners). **e**, Examples of coverage of the effective arena (in the corresponding reference frame) by fields of different cell types (indicated). Scale bar: 20 cm. **f**, Distribution of field coverage across sessions for all cell types (mean ± s.e.m.; n = 18 sessions.). **g**, Smoothed heatmaps of field density indicate that the fields of selfPCs and socialPCs accumulate close to the walls of the arena, while those of alloSVCs and egoSVCs are homogenously distributed across the effective arena. Scale bar: 20 cm. **h**, Diagram showing overlap index (color coded) and indicating p-value of the associated Binomial test for all pairs of cell types. In box plots, the central line indicates the median, and the bottom and top edges of the box mark the interquartile range. Whiskers extend from -1.5 × i.q.r. to +1.5 × i.q.r. from the closest quartile, where i.q.r. is the interquartile range. Black dots mark outliers. Applied to all supplementary figures. Source data are provided as a Source Data file.

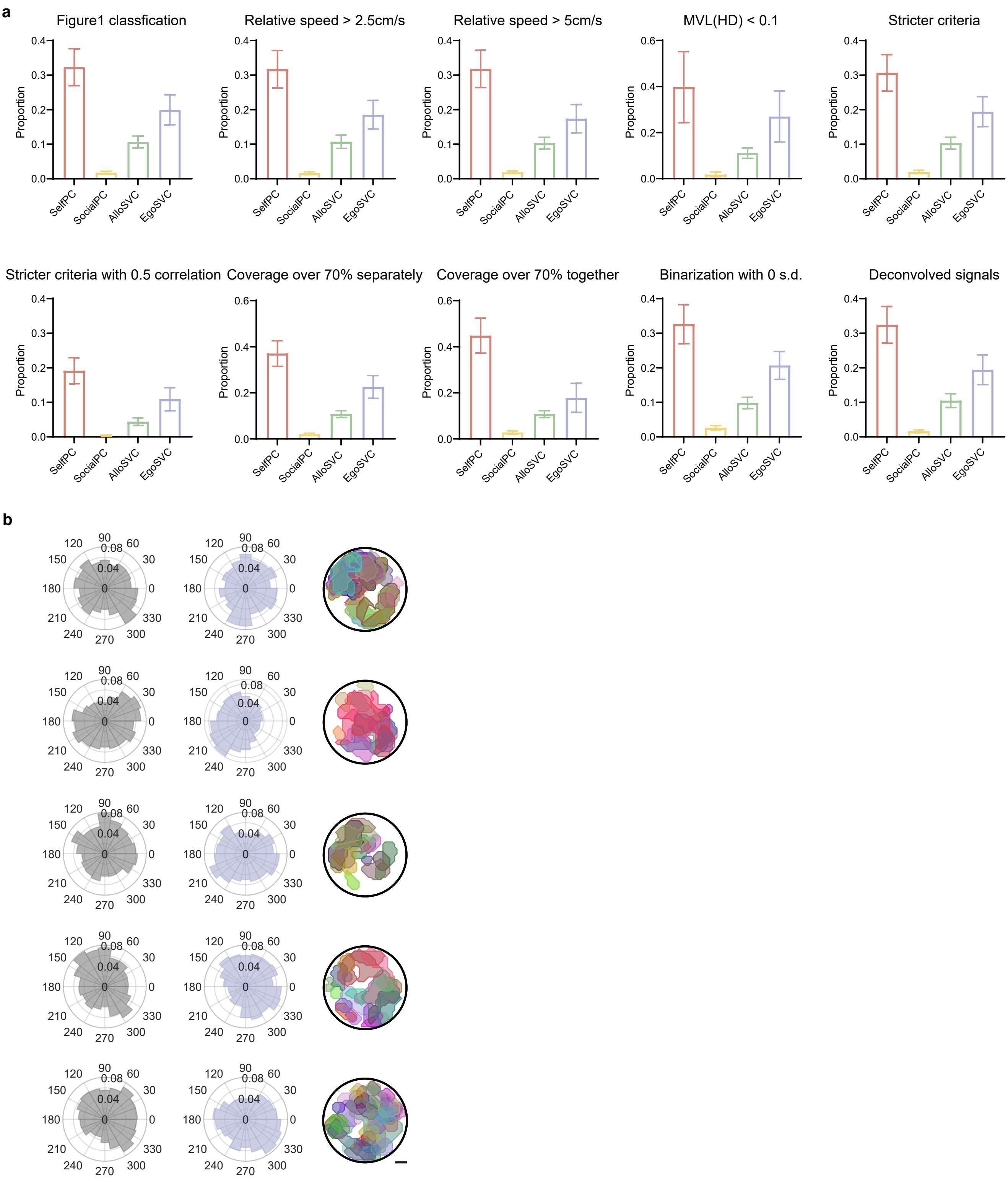
a
Figure1 classfication
Relative speed > 2.5cm/s
Relative speed > 5cm/s
MVL(HD) < 0.1
Stricter criteria
Stricter criteria with 0.5 correlation
Coverage over 70% separately
Coverage over 70% together
Binarization with 0 s.d.
Deconvolved signals
Proportion
SelfPC
SocialPC
AlloSVC
EgoSVC
b

Supplementary Figure 4. Social-vector cells are consistently found under different criteria.

a, Fraction of cells classified as belonging to each cell type under various criteria, from left to right and top to bottom (mean ± s.e.m.). First, classification obtained with the criteria applied in Figure 1. Second, removing episodes in which both animals execute similar movements by considering only episodes of relative speed higher than 2.5 cm s^{-1} (effectively removing 12 ± 2 % of data from each session). Third, a similar approach but using a cutoff value of 5 cm s^{-1} (effectively removing 26 ± 3 % of data from each session). Fourth, selecting only sessions with very homogeneous coverage of head direction by the imaged mouse (mean vector length < 0.1; 5 out of 18 sessions; see **b**). Fifth, imposing a set of stricter criteria on cell classification: mean event rate above 0.1 Hz and a detectable field of at least 25 spatial bins (5 × 5 bins; side of square bins: 2 cm for selfPCs and socialPCs or 4 cm for alloSVCs and egoSVCs). Sixth: similar stricter criteria but adding a minimum correlation between halves of the session of 0.5. Seventh, considering for each cell type classification only sessions with coverage above 70 %. Eighth, considering only sessions in which coverage was above 70 % for all coordinate systems simultaneously (8 out of 18 sessions; see examples in Supplementary Fig. 5**b**). Ninth, binarizing deconvoluted calcium data with a threshold of 0 instead of 3 standard deviations. Tenth, using deconvolved signals without binarization. Note that the relative proportion of cell types is roughly maintained. **b**, For all sessions (one per animal; rows) with very homogeneous cover of head direction by the imaged mouse (mean vector length < 0.1), angular distribution of head direction (left subpanel), angular distribution of egoSVC direction (center subpanel) and egoSVC fields (right subpanel). Scale bar: 20 cm. Source data are provided as a Source Data file.

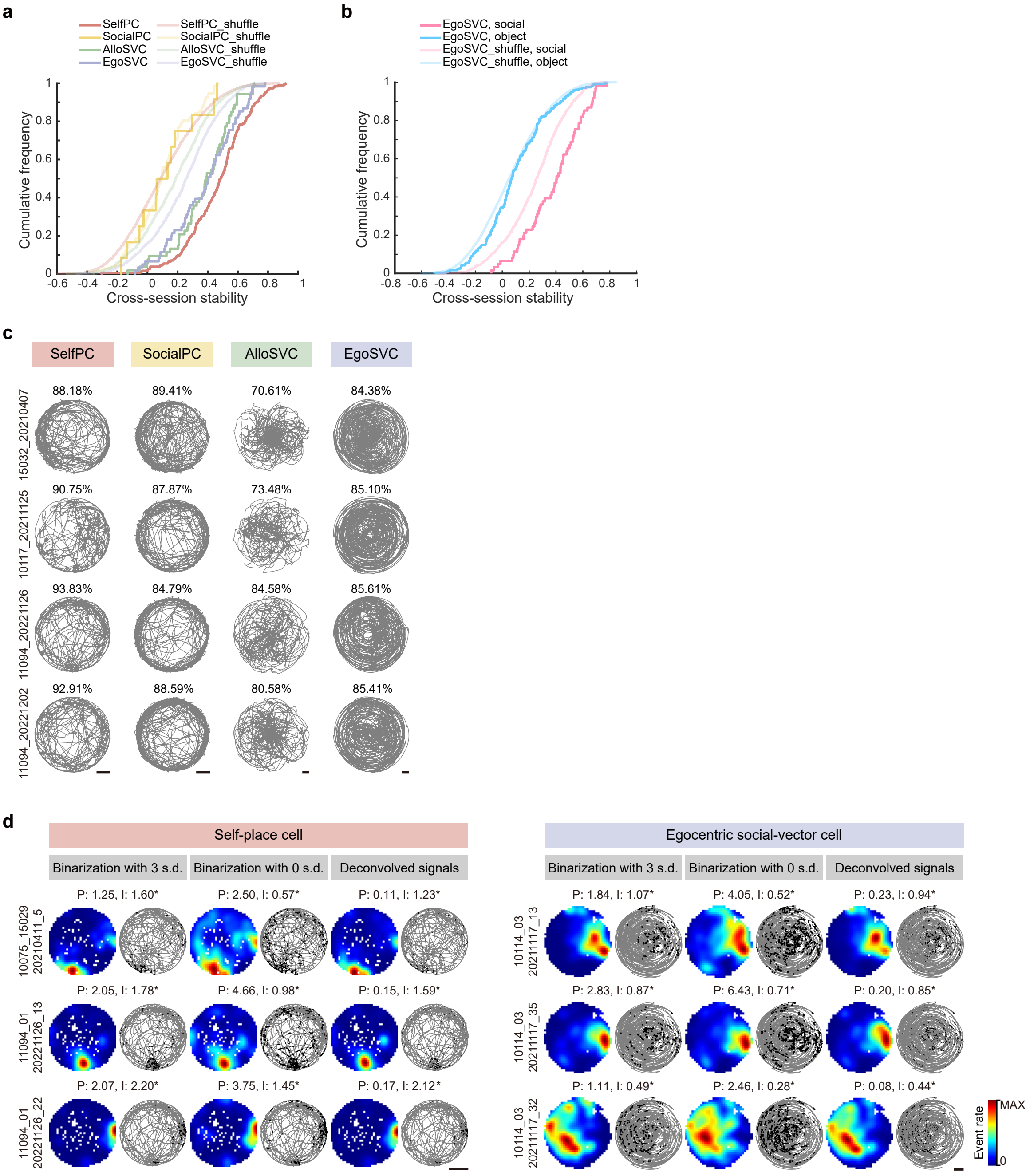
a
SelfPC
SocialPC
AlloSVC
EgoSVC
SelfPC_shuffle
SocialPC_shuffle
AlloSVC_shuffle
EgoSVC_shuffle
Cumulative frequency
Cross-session stability
b
EgoSVC, social
EgoSVC, object
EgoSVC_shuffle, social
EgoSVC_shuffle, object
Cumulative frequency
Cross-session stability
c
SelfPC
SocialPC
AlloSVC
EgoSVC
15032_20210407
88.18%
89.41%
70.61%
84.38%
10117_20211125
90.75%
87.87%
73.48%
85.10%
11094_20221126
93.83%
84.79%
84.58%
85.61%
11094_20221202
92.91%
88.59%
80.58%
85.41%
d
Self-place cell
Binarization with 3 s.d.
Binarization with 0 s.d.
Deconvolved signals
10075_15029 20210411_5
P: 1.25, I: 1.60*
P: 2.50, I: 0.57*
P: 0.11, I: 1.23*
11094_01 20221126_13
P: 2.05, I: 1.78*
P: 4.66, I: 0.98*
P: 0.15, I: 1.59*
11094_01 20221126_22
P: 2.07, I: 2.20*
P: 3.75, I: 1.45*
P: 0.17, I: 2.12*
Egocentric social-vector cell
Binarization with 3 s.d.
Binarization with 0 s.d.
Deconvolved signals
10114_03 20211117_13
P: 1.84, I: 1.07*
P: 4.05, I: 0.52*
P: 0.23, I: 0.94*
10114_03 20211117_35
P: 2.83, I: 0.87*
P: 6.43, I: 0.71*
P: 0.20, I: 0.85*
10114_03 20211117_32
P: 1.11, I: 0.49*
P: 2.46, I: 0.28*
P: 0.08, I: 0.44*
Event rate
MAX
0

Supplementary Figure 5. Considerations around stability, coverage or pre-processing of data do not explain social-vector cell activity.

a, For two control animals, distribution of cross-session stability (two consecutive 10 min sessions with 5 min rest) for different cell types (color coded) and corresponding shuffled distribution (pale colors) (observed vs. shuffle Mann Whitney test, two tailed; selfPC, socialPC, alloSVC, egoSVC: Mann-Whitney U = 460709, 7026, 71402, 122598; Cliff's Delta = 0.73, 0.02, 0.49, 0.34; p = 8.9×10^{-66}, 0.89, 6.9×10^{-10}, 4.4×10^{-6}). We found a significant difference across categories (Kruskal-Wallis test, H = 28.58, p = 2.7×10^{-6}) due to a lower cross-session correlation of socialPCs relative to all other categories (Dunn's multiple comparisons test, all p-values < 0.02) and of alloSVCs relative to selfPCs (p = 0.04). **b**, For animals shown in **a**, comparison of egoSVC cross-session stability for two sessions with a social partner as in **a** and two similar sessions with an object that was moved to a new location between sessions. Cross-session stability for object-egoSVCs was not significantly higher than its corresponding shuffled distribution (two-tailed Mann-Whitney test, U = 2559172, Cliff's Delta = 0.07, p = 0.053), and significantly lower than cross-session stability for regular egoSVCs (two-tailed Mann-Whitney test U = 2738, Cliff's Delta = 0.62, p = 4.1×10^{-15}). **c**, Examples of coverage (quantification indicated) in all coordinate systems (columns) for 4 of the 8 sessions (one per row) with simultaneous coverage above 70 %. Scale bar: 20 cm. **d**, Representative examples (one per row) of rate and trajectory maps of selfPCs (left) and egoSVCs (right) with deconvolved calcium data binarized with a threshold of 3 standard deviations, as in the rest of this work (left subpanels), using a binarization threshold of 0 standard deviations above the mean (center subpanels) and directly using the deconvolved signal with no binarization (right subpanels). Scale bar: 20 cm. Source data are provided as a Source Data file.

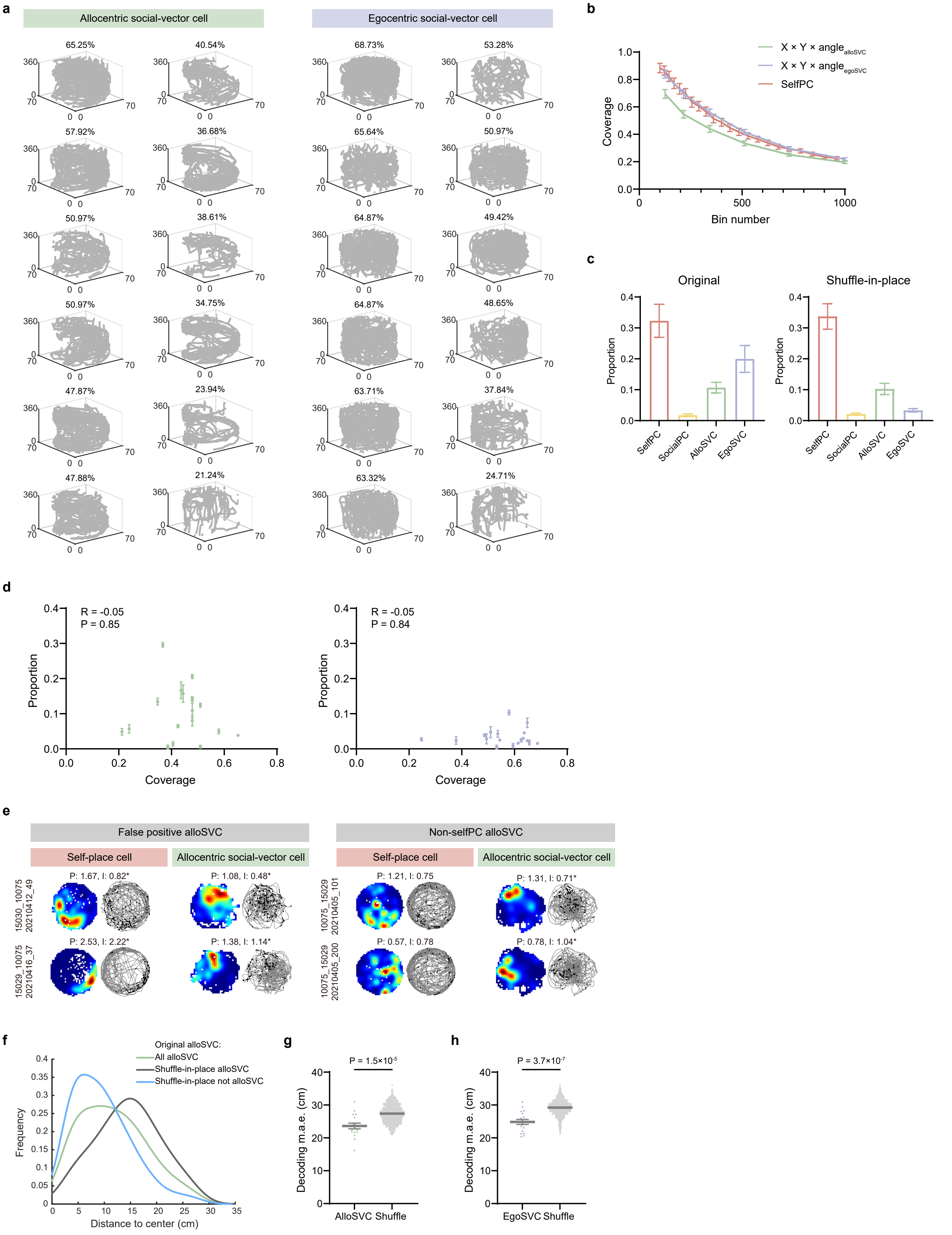
a
Allocentric social-vector cell
Egocentric social-vector cell
65.25%
40.54%
57.92%
36.68%
50.97%
38.61%
50.97%
34.75%
47.87%
23.94%
47.88%
21.24%
68.73%
53.28%
65.64%
50.97%
64.87%
49.42%
64.87%
48.65%
63.71%
37.84%
63.32%
24.71%
b
X × Y × angle alloSVC
X × Y × angle egoSVC
SelfPC
Coverage
Bin number
c
Original
Shuffle-in-place
Proportion
SelfPC
SocialPC
AlloSVC
EgoSVC
d
R = -0.05
P = 0.85
R = -0.05
P = 0.84
Proportion
Coverage
e
False positive alloSVC
Non-selfPC alloSVC
Self-place cell
Allocentric social-vector cell
15030_10075 20210412_49
15029_10075 20210416_37
10075_15029 20210405_101
10075_15029 20210405_200
P: 1.67, I: 0.82*
P: 1.08, I: 0.48*
P: 2.53, I: 2.22*
P: 1.38, I: 1.14*
P: 1.21, I: 0.75
P: 1.31, I: 0.71*
P: 0.57, I: 0.78
P: 0.78, I: 1.04*
f
Original alloSVC:
All alloSVC
Shuffle-in-place alloSVC
Shuffle-in-place not alloSVC
Frequency
Distance to center (cm)
g
P = 1.5×10⁻⁵
Decoding m.a.e. (cm)
AlloSVC Shuffle
h
P = 3.7×10⁻⁷
Decoding m.a.e. (cm)
EgoSVC Shuffle

Supplementary Figure 6. Geometric factors cause false positives in the classification of alloSVCs.

a, Trajectory of each mouse pair in selfPC (x and y coordinates) and alloSVC (left panel) or egoSVC (right panel) angle (z coordinate). Fraction of covered bins indicated. **b**, Distribution across sessions (mean ± s.e.m.) of coverage as a function of the total bin number for selfPC × alloSVC angle (green), selfPC × egoSVC angle (purple) and selfPC (red). **c**, Original classification (left; Supplementary Fig. 4**a**) and classification of cells after shuffling-in-place each cell 10 times (right). only 42% of alloSVCs and 92% of egoSVCs had information rate higher than the 95th percentile of their corresponding shuffle-in-place distributions (1000 shuffles). Only 57.1 % of alloSVCs had shuffles-in-place that were not alloSVCs (96.5 % for egoSVCs). While the initial classification suggested that alloSVCs constitute on average 10.7 % of all cells, incorporating the supplementary criterion of more than half of shuffles-in-place not being classified as alloSVC reduces the proportion to 6 % of cells. **d**, Cross-session correlation between the proportion of shuffles-in-place classified as alloSVC (left) and egoSVC (right) and coverage across sessions (mean ± s.e.m.). **e**, Examples of a systematic geometrical bias responsible of false positives in the classification of alloSVCs (left) and counterexamples (non-selfPC alloSVC, right). Each row shows the selfPC (left) and alloSVC (right) map of a cell. If a selfPC field is close to the left border activity is biased to appear in the right part of the alloSVC map. **f**, Distribution of the distance of the selfPC center of mass to the center of the arena for all alloSVCs (green). Similar plots for the subgroup that has a majority of shuffles-in-place also classified as alloSVC (i.e. false positives, grey) and those not classified as alloSVC (blue). **g**, Decoding error in alloSVC coordinates is lower than the shuffled distribution when only alloSVCs with shuffles-in-place not classified as alloSVCs are used (mean ± s.e.m., Mann Whitney test, two tailed. Mann-Whitney U = 5637, Cliff's Delta = -0.61, $p = 1.5\times10^{-5}$). **h**, Similar to **g** but for egoSVCs (Mann Whitney test, two tailed. Mann-Whitney U = 4104, Cliff's Delta = -0.72, $p = 3.7\times10^{-7}$). Source data are provided as a Source Data file.

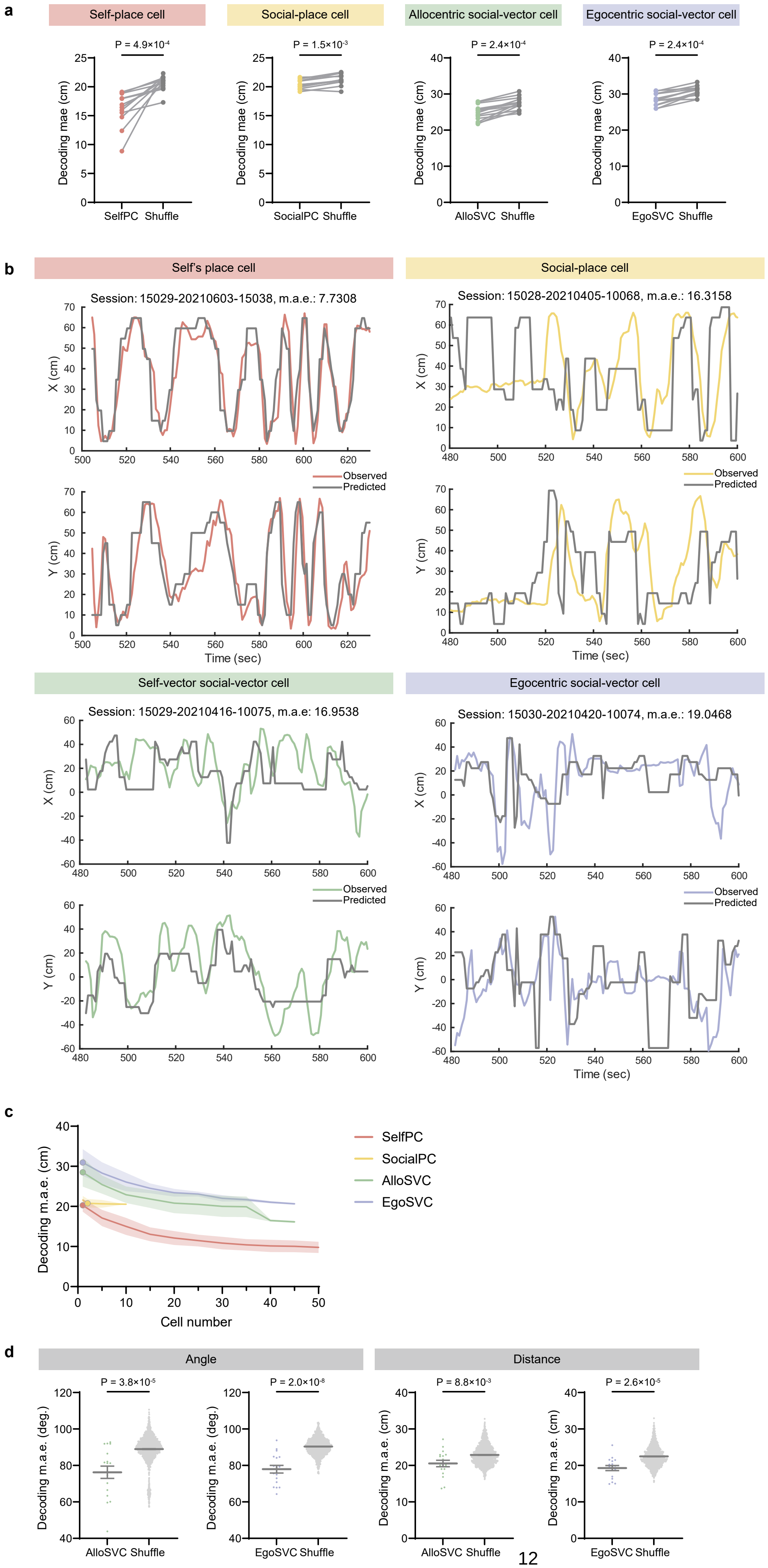
a
Self-place cell
Social-place cell
Allocentric social-vector cell
Egocentric social-vector cell
P = 4.9×10-4
P = 1.5×10-3
P = 2.4×10-4
P = 2.4×10-4
Decoding mae (cm)
SelfPC Shuffle
SocialPC Shuffle
AlloSVC Shuffle
EgoSVC Shuffle
b
Self's place cell
Social-place cell
Session: 15029-20210603-15038, m.a.e.: 7.7308
Session: 15028-20210405-10068, m.a.e.: 16.3158
X (cm)
Y (cm)
Observed
Predicted
Time (sec)
Self-vector social-vector cell
Egocentric social-vector cell
Session: 15029-20210416-10075, m.a.e: 16.9538
Session: 15030-20210420-10074, m.a.e.: 19.0468
c
Decoding m.a.e. (cm)
Cell number
SelfPC
SocialPC
AlloSVC
EgoSVC
d
Angle
Distance
P = 3.8×10-5
P = 2.0×10-8
P = 8.8×10-3
P = 2.6×10-5
Decoding m.a.e. (deg.)
Decoding m.a.e. (cm)
AlloSVC Shuffle
EgoSVC Shuffle

Supplementary Figure 7. Examples of decoding and statistics by mouse in different reference frames.

a, Error in decoding for different cell types, as in Fig. 1**j**, but grouping data by mouse (one dot per mouse; Wilcoxon matched-pairs signed rank test, p-value indicated; from left to right, n = 12, 12, 13, 13 mice. W = -78, -74, -91, -91; Cliff's Delta = -0.92, -0.45, -0.56, -0.73). **b**, Examples of predicted (grey) and observed (color) trajectory belonging to the test data for different cell types (indicated). **c**, Error in decoding as a function of cell number for all cell types (color coded), using all sessions in Fig. 1 with at least that number of cells available (mean ± s.d.). Dots: for each cell type, number of cells necessary to have an error significantly lower than the one obtained for shuffled data. **d**, Error in decoding angle or distance independently, for alloSVCs, egoSVCs and the corresponding shuffled data (18 sessions in Fig. 1; Mann Whitney test, two tailed; from left to right, Mann-Whitney U = 6071, 3040, 9123, 5888; Cliff's Delta = -0.58, -0.79, -0.37, -0.59; p-value indicated). Source data are provided as a Source Data file.

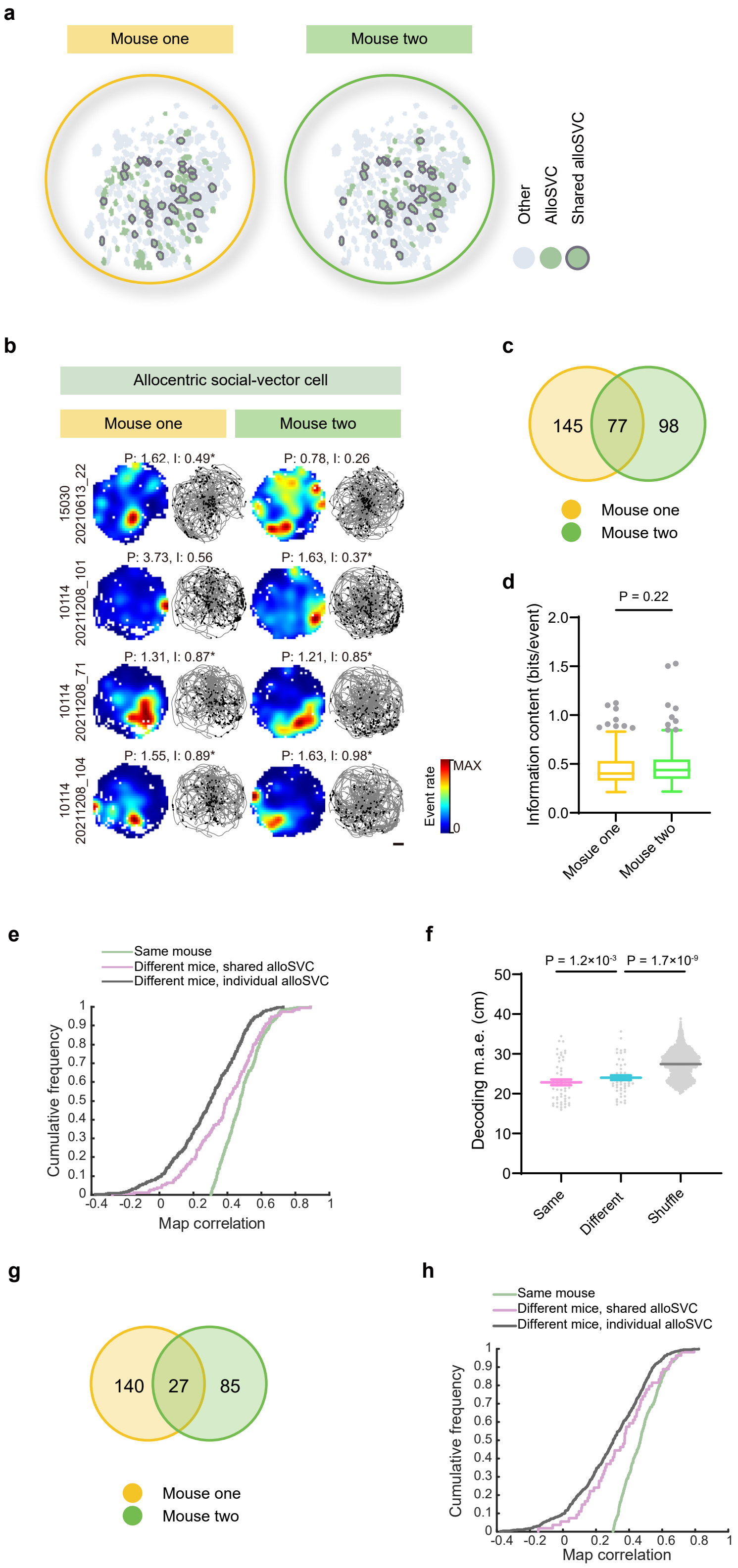

a
Mouse one
Mouse two
Other
AlloSVC
Shared alloSVC
b
Allocentric social-vector cell
Mouse one
Mouse two
15030 20210613_22
P: 1.62, I: 0.49*
P: 0.78, I: 0.26
10114 20211208_101
P: 3.73, I: 0.56
P: 1.63, I: 0.37*
10114 20211208_71
P: 1.31, I: 0.87*
P: 1.21, I: 0.85*
10114 20211208_104
P: 1.55, I: 0.89*
P: 1.63, I: 0.98*
Event rate
MAX
0
c
145
77
98
Mouse one
Mouse two
d
P = 0.22
Information content (bits/event)
Mosue one
Mouse two
e
Same mouse
Different mice, shared alloSVC
Different mice, individual alloSVC
Cumulative frequency
Map correlation
f
P = 1.2×10⁻³
P = 1.7×10⁻⁹
Decoding m.a.e. (cm)
Same
Different
Shuffle
g
140
27
85
Mouse one
Mouse two
h
Same mouse
Different mice, shared alloSVC
Different mice, individual alloSVC
Cumulative frequency
Map correlation

Supplementary Figure 8. Allocentric social-vector cells code mouse identity.

Panels similar to Fig. 2 but analyzing alloSVCs. **a**, Representative example of the distribution inside the field of view of cells coding for mouse 1 (left, orange, mouse with a LED), mouse 2 (right, green) or both (grey borders). **b**, Examples of rate maps and trajectory of alloSVCs using coordinates for mouse 1 (left) or mouse 2 (right). Scale bar: 20 cm. **c**, Venn diagram of alloSVCs responding to each mouse (overlap index: 1.78, two-tailed binomial test $p = 1.64 \times 10^{-6}$). **d**, Information content for cells classified as alloSVC for mouse 1 (left) or 2 (right) (median ± i.q.r., Mann Whitney test, two tailed, Mann-Whitney U = 18054, Cliff's Delta = -0.07, p = 0.22). **e**, Cumulative distribution of correlation between maps corresponding to the first and second halves of a session, using the relative position of the same (green) or a different mouse (pink and grey), for cells that were classified as alloSVC for both (pink) or only one mouse (grey) (Kruskal-Wallis test, H = 223.2, $p = 3.45 \times 10^{-49}$; Dunn's multiple comparisons test, all p-values $< 2.0 \times 10^{-7}$). **f**, Cross-validated error for decoding the relative position of a mouse with a decoder trained with data corresponding to the same (pink) or the other (blue) mouse, together with the shuffled distribution (grey) (mean ± s.e.m., one dot per session, 53 sessions; two-tailed Wilcoxon matched-pairs signed rank tests for same vs different mouse: W = 715, Cliff's Delta = -0.19, $p = 1.2 \times 10^{-3}$; two tailed Mann Whitney test for different mouse vs shuffle: Mann-Whitney U = 76096, Cliff's Delta = -0.49, $p = 1.7 \times 10^{-9}$) **g**, Venn diagram as **c** but for alloSVCs with shuffles-in-place not classified as alloSVC (see Supplementary Fig. 6, overlap index: 1.30, two-tailed binomial test p = 0.19). **h**, As **e** but for alloSVCs with shuffles-in-place not classified as alloSVC (see Supplementary Fig. 6; Kruskal-Wallis test, H = 135.5, $p = 3.3 \times 10^{-30}$. Dunn's multiple comparisons test, p(same vs shared) = 6.9×10^{-5}, p(same vs individual) = 7.9×10^{-31}, p(shared vs individual) = 0.22). Source data are provided as a Source Data file.

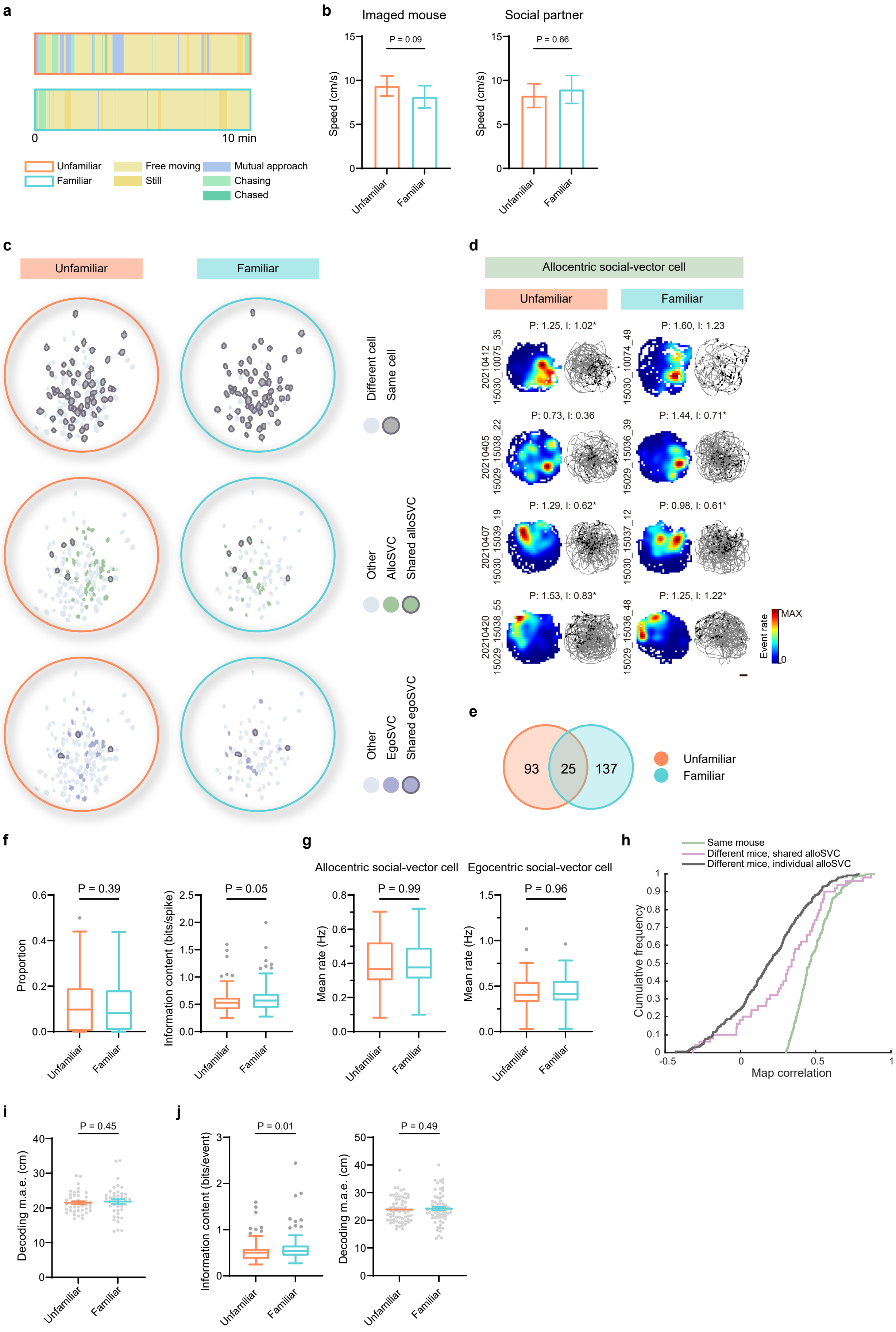
a
0
10 min
Unfamiliar
Familiar
Free moving
Still
Mutual approach
Chasing
Chased
b
Imaged mouse
Social partner
P = 0.09
P = 0.66
Speed (cm/s)
Unfamiliar
Familiar
c
Unfamiliar
Familiar
Different cell
Same cell
Other
AlloSVC
Shared alloSVC
Other
EgoSVC
Shared egoSVC
d
Allocentric social-vector cell
Unfamiliar
Familiar
P: 1.25, I: 1.02*
P: 1.60, I: 1.23
20210412 15030_10075_35
15030_10074_49
P: 0.73, I: 0.36
P: 1.44, I: 0.71*
20210405 15029_15038_22
15029_15036_39
P: 1.29, I: 0.62*
P: 0.98, I: 0.61*
20210407 15030_15039_19
15030_15037_12
P: 1.53, I: 0.83*
P: 1.25, I: 1.22*
20210420 15029_15038_55
15029_15036_48
Event rate
MAX
0
e
93
25
137
Unfamiliar
Familiar
f
P = 0.39
P = 0.05
Proportion
Information content (bits/spike)
g
Allocentric social-vector cell
Egocentric social-vector cell
P = 0.99
P = 0.96
Mean rate (Hz)
h
Same mouse
Different mice, shared alloSVC
Different mice, individual alloSVC
Cumulative frequency
Map correlation
i
P = 0.45
Decoding m.a.e. (cm)
j
P = 0.01
P = 0.49
Information content (bits/event)
Decoding m.a.e. (cm)

Supplementary Figure 9. No modulation of allocentric social vector cell activity by familiarity.

a, Representative example of interactions during an unfamiliar (top) and familiar (bottom) session (type of interaction color-coded). **b**, Running speed of imaged mouse (left; mean ± s.e.m., n = 8 mice pairs; two-tailed paired t test: t = 1.986, df = 7, p = 0.09) and conspecific (right; mean ± s.e.m., n = 8 mice pairs; two-tailed paired t test: t = 0.4639, df = 7, p = 0.66). **c**, Example of cross-session cell tracking for unfamiliar (left column) and familiar (right column) sessions (shared and non-shared social-vector cells indicated). **d**, Representative alloSVC examples for unfamiliar (left) or familiar (right) sessions (panels arranged as in Fig. 1e). Scale bar: 20 cm. **e**, Venn diagram of alloSVCs for each mouse (255 out of 1948 cells, 8 mice; overlap index: 2.55, two-tailed Binomial test, $p = 3.3\times10^{-5}$). **f**, Proportion of alloSVCs (left, median ± i.q.r.) and information content (right, median ± i.q.r.) for unfamiliar and familiar sessions (106 sessions, two-tailed Wilcoxon matched-pairs signed rank test, W = -436, 649; Cliff's Delta = -0.05, -0.11; p-values indicated). **g**, As **f** but showing mean calcium event rate for alloSVCs (left) and egoSVCs (right) (median ± i.q.r., two-tailed Wilcoxon matched-pairs signed rank test, W = -1, 28; Cliff's Delta = 2.6×10^{-3}, -0.02; p-values indicated). **h**, Cumulative distribution of correlation between maps corresponding to the first and second halves of a session (plots as in Fig. 3**g**; Kruskal-Wallis test, H = 63.38, $p = 1.7\times10^{-14}$; Dunn's multiple comparisons test, all p-values < 0.002). **i**, Cross-validated alloSVC decoding error for unfamiliar and familiar sessions (mean ± s.e.m., one dot per session; two-tailed Wilcoxon matched-pairs signed rank test, W = 228, Cliff's Delta = -0.05, p = 0.45). **j**, For alloSVCs with shuffles-in-place not classified as alloSVC (see Supplementary Fig. 6), information content (left, median ± i.q.r., two-tailed Wilcoxon matched-pairs signed rank test, W = 765, Cliff's Delta = -0.15, p = 0.01) and cross-validated decoding error (right, mean ± s.e.m., one dot per session; two-tailed Wilcoxon matched-pairs signed rank test, W = 210, Cliff's Delta = -0.04, p = 0.49) for unfamiliar and familiar sessions. Source data are provided as a Source Data file.

a

Analysis of variance

Source	Sum Sq.	d.f.	Mean Sq.	F	Prob>F
Familiarity	0.9	1	0.92	4.74×10^{-3}	0.95
Interaction	36.1	1	36.06	0.19	0.67
Distance	57227.9	6	9537.99	49.41	1.26×10^{-59}
Error	1217738.3	6308	193.05		
Total	1279481.4				

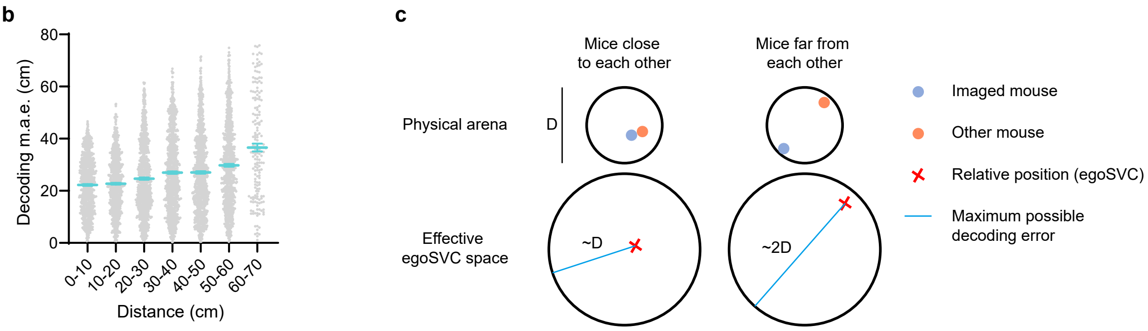

Supplementary Figure 10. Closer distance explains low decoding error for unfamiliar sessions

a, ANOVA analysis to explain variability in cross-validated decoding error within sessions (pool of 5 unfamiliar and 5 familiar sessions) using familiarity, interaction (whether or not mice were interacting at a given timestamp) and distance between mice as independent variables. **b**, For data in Fig. 3**i**, decoding error as a function of distance between mice (mean ± s.e.m.). The improvement in decoding error for unfamiliar mice is explained by the greater amount of time spent at shorter distances. This is consistent with a bias in egoSVC decoding error for mice that are far apart related to purely geometrical reasons. **c**, Schematic explanation of the bias in egoSVC decoding error for mice that are far apart. Mice very close to each other in physical space (top, left) have a relative position in the egoSVC effective arena close to the center (bottom, left). In this situation, the maximum error for a decoder is the radius of the effective arena, equal to the diameter of the physical arena. Mice in opposite ends of the physical arena (top, right) have a relative position in the egoSVC effective arena close to the edge (bottom, right). In this situation, the maximum error for a decoder is the diameter of the effective arena, equal to twice the diameter of the physical arena. Source data are provided as a Source Data file.

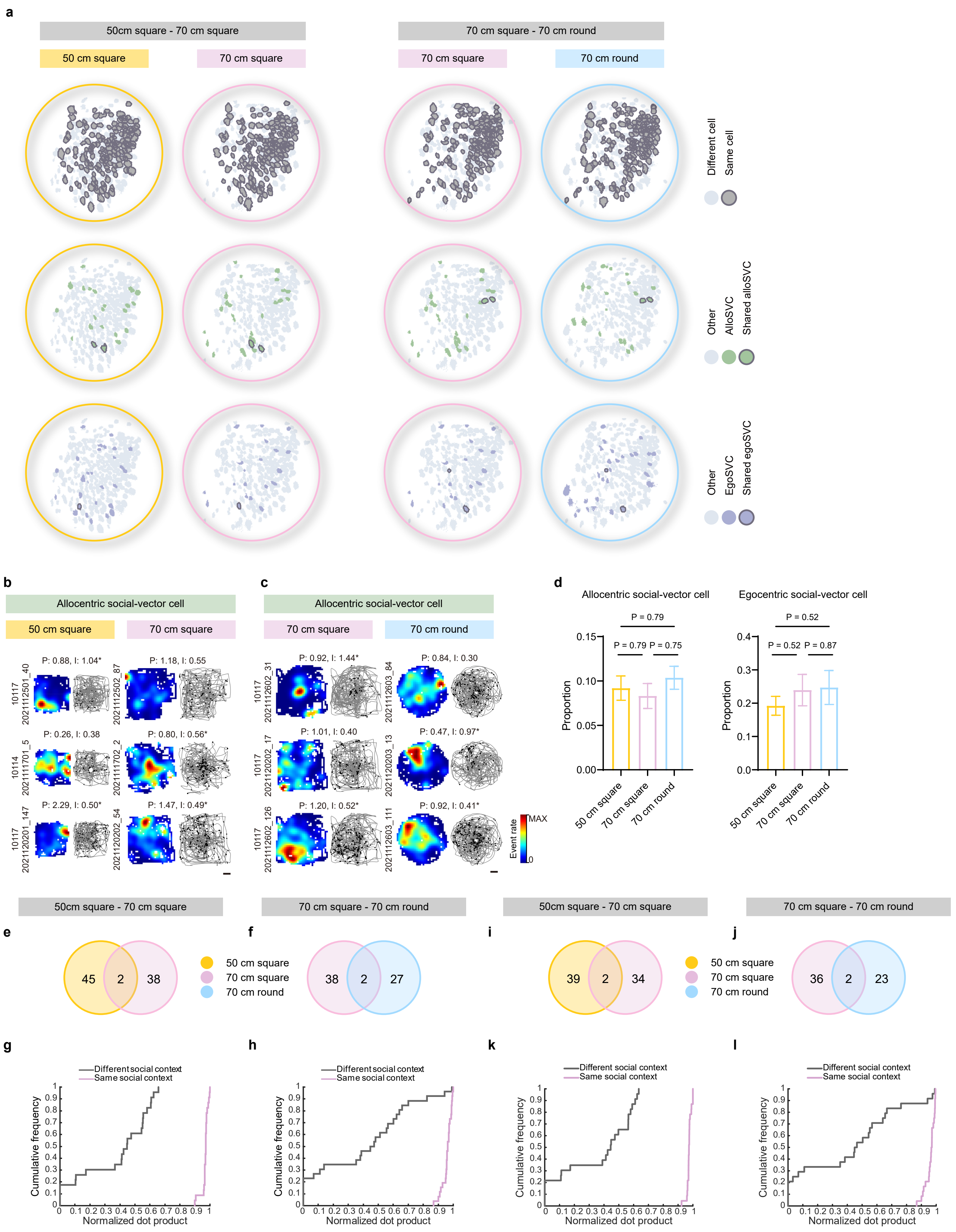
a
50cm square - 70 cm square
70 cm square - 70 cm round
50 cm square
70 cm square
70 cm round
Different cell
Same cell
Other
AlloSVC
Shared alloSVC
EgoSVC
Shared egoSVC
b
c
Allocentric social-vector cell
d
Allocentric social-vector cell
Egocentric social-vector cell
P = 0.79
P = 0.75
P = 0.52
P = 0.87
Proportion
Event rate
MAX
e
f
i
j
g
h
k
l
Different social context
Same social context
Cumulative frequency
Normalized dot product

Supplementary Figure 11. Allocentric social-vector representations are not context-invariant.

a, As in Supplementary Fig. 9**c**, example of tracked cells for the size (columns 1 and 2) and shape (columns 3 and 4) experiments. **b-c**, Representative examples (as in Fig. 1**e**) of cells classified as alloSVC (asterisk) in at least one of two consecutive sessions, testing for effects of size (**a**) or shape (**b**). Scale bar: 20 cm. **d**, Proportion of alloSVCs (left) and egoSVCs (right) classified in the three different environments (mean ± s.e.m., Mixed-effected model, F (1.910, 75.45) = 0.5682, p = 0.56, p-values of Holm-Šídák's multiple comparisons test indicated). **e-f**, Venn diagrams representing overlaps between subpopulations of cells classified as alloSVC in consecutive sessions (4 imaged mice, 1003 imaged cells; overlap indexes sessions 1 vs. 2: 1.07, Binomial test p: 0.71; sessions 2 vs. 3: 1.73, Binomial test p: 0.32). **g-h**, Cumulative distribution of the normalized dot product, quantifying the similarity of the mean firing rate of alloSVCs across halves in the same context (violet) or across contexts (grey) of different size (f) or shape (g). Halves of sessions in the same context were more similar than in different contexts (27 sets of paired sessions, two-tailed Mann Whitney test sessions 1 vs. 2: Mann-Whitney U = 0, Cliff's Delta = 1, p = 2.4×10^{-13}; sessions 2 vs. 3: Mann-Whitney U = 33, Cliff's Delta = 0.90, p = 2.2×10^{-10}). **i-j**, As **e-f** but for alloSVCs with shuffles-in-place not classified as alloSVC (see Supplementary Fig. 6), Venn diagrams representing overlaps in consecutive sessions (overlap indexes sessions 1 vs. 2: 1.36, Binomial test p: 0.66; sessions 2 vs. 3: 2.11, Binomial test p: 0.24). **k-l**, As **g-h** but for alloSVCs with shuffles-in-place not classified as alloSVC (see Supplementary Fig. 6; two-tailed Mann Whitney test sessions 1 vs. 2: Mann-Whitney U = 0, Cliff's Delta = 1, p = 2.4×10^{-13}; sessions 2 vs. 3: Mann-Whitney U = 40, Cliff's Delta = 0.86, p = 1.3×10^{-8}). Source data are provided as a Source Data file.

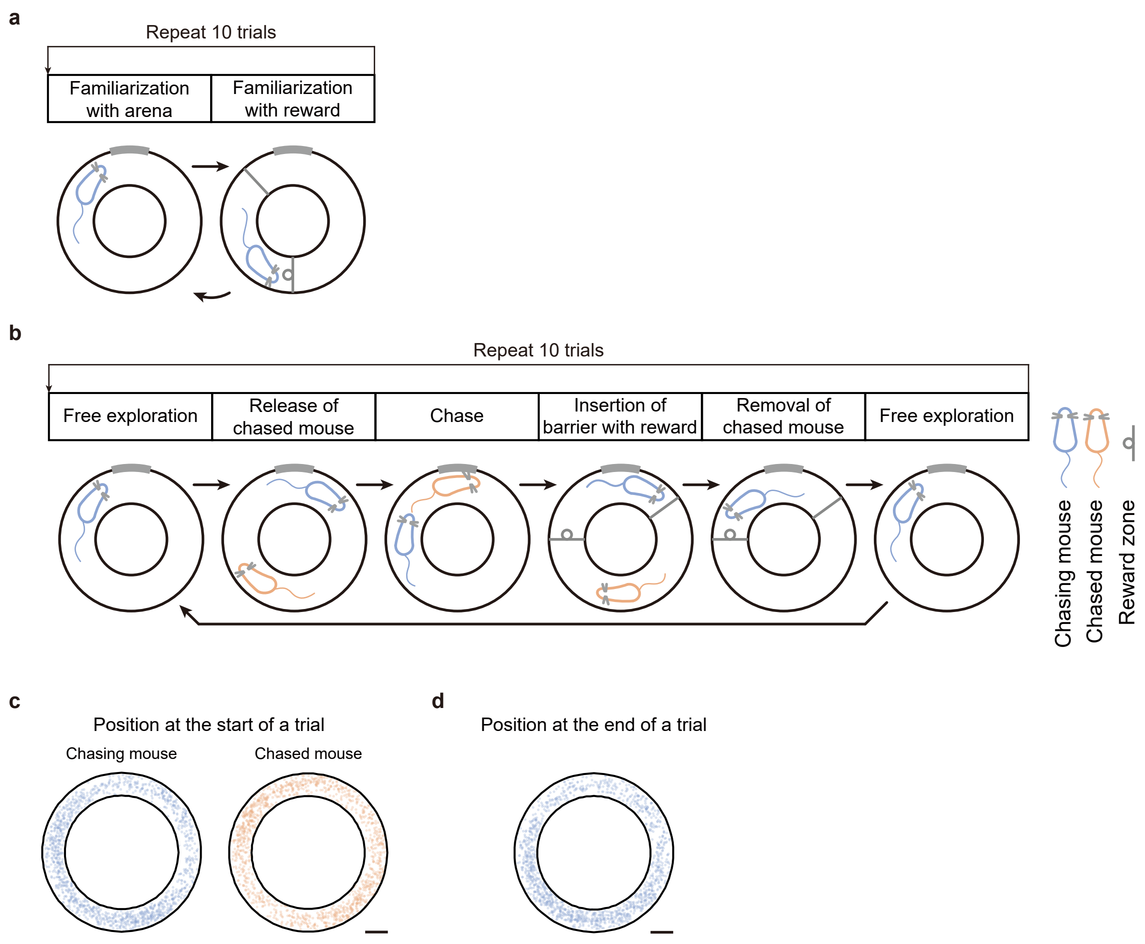

Supplementary Figure 12. Behavioral training in the novel pursuit task

a, Before proper training, all mice are familiarized with the annular maze. In addition to free navigation in the maze, the mouse that will be trained to closely follow a conspecific (mouse A) is familiarized with reward events, during which a paperboard barrier is inserted and a reward placed on the barrier (3 days, 10 reward events per session). **b**, Pursuit task. Mouse A is placed in the annular maze. After a period of 1-2 minutes, a second mouse (B) is placed in the arena at a random position. Whenever mouse A closely follows mouse B, the barrier containing a reward for mouse A is inserted. Mouse B is removed and put in a pedestal, where it also receives a reward. The barrier is removed and mouse A is left to freely explore the maze again. The pursuit task is repeated 10 times in each session. **c**, Positions of mice A (left) and B (right) at the beginning of a pursuit trial (one dot per trial). Scale bar: 10 cm. **d**, Positions of mouse A when the barrier is inserted for reward delivery. Scale bar: 10 cm.

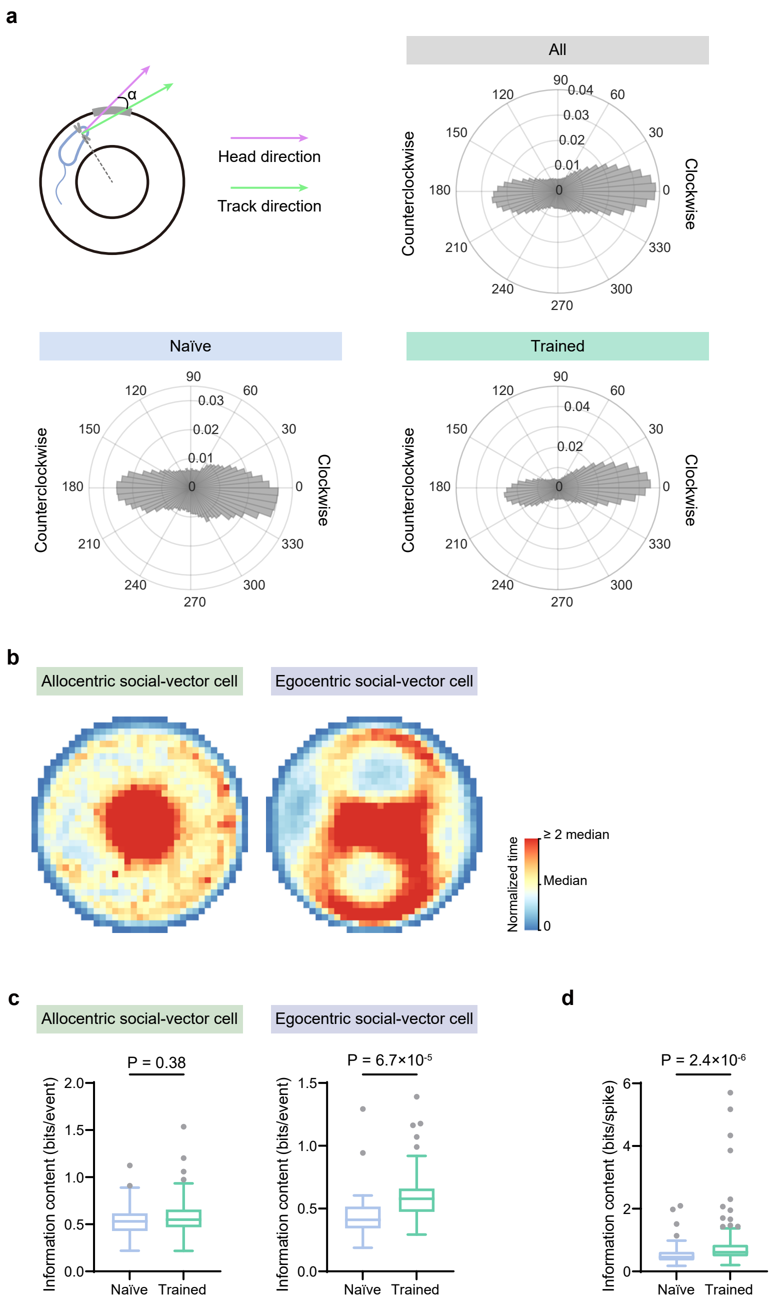
a
Head direction
Track direction
All
Naïve
Trained
Counterclockwise
Clockwise
b
Allocentric social-vector cell
Egocentric social-vector cell
Normalized time
≥ 2 median
Median
0
c
Allocentric social-vector cell
Egocentric social-vector cell
P = 0.38
P = 6.7×10-5
Information content (bits/event)
Naïve
Trained
d
P = 2.4×10-6
Information content (bits/spike)
Naïve
Trained

Supplementary Figure 13. Increased information for trained animals is not explained by geometric factors.

a, Figure 6 represents egoSVC data on a single variable under the approximation that head direction is mostly tangential to the track (upper left panel). For all (upper right panel), naïve (lower left panel) and trained (lower right panel) mice, angular difference between head and track direction, showing prominent maxima corresponding to clockwise (0°) and counterclockwise (180°) running. **b**, For all sessions with trained mice, cumulative occupancy of the alloSVC (left) and egoSVC (right) effective arenas. In the egoSVC case, a figure 8 emerges, the top (bottom) loop corresponding to counterclockwise (clockwise) trajectories. In a control analysis to rule out that stereotyped patterns of behavior explained the difference in egoSVC information observed in Figures 5 and 6, only bins with occupancy levels above the median were selected (50 %) of bins. **c**, Information content as in Figure 5**e** for alloSVCs (left) and egoSVCs (right) in naïve and trained mice using only spatial bins selected in **b** (median ± i.q.r., two-tailed Mann Whitney test, Mann-Whitney U = 1019, 526, Cliff's Delta = -0.11, -0.51, p = 0.38, 6.7×10^{-5}). **d**, Information content for egoSVC angle maps, as in Figure 6**c**, but using only spatial bins selected in **b** (median ± i.q.r., two-tailed Mann Whitney test, Mann-Whitney U = 4354, Cliff's Delta = -0.37, p = 2.4×10^{-6}). Source data are provided as a Source Data file.

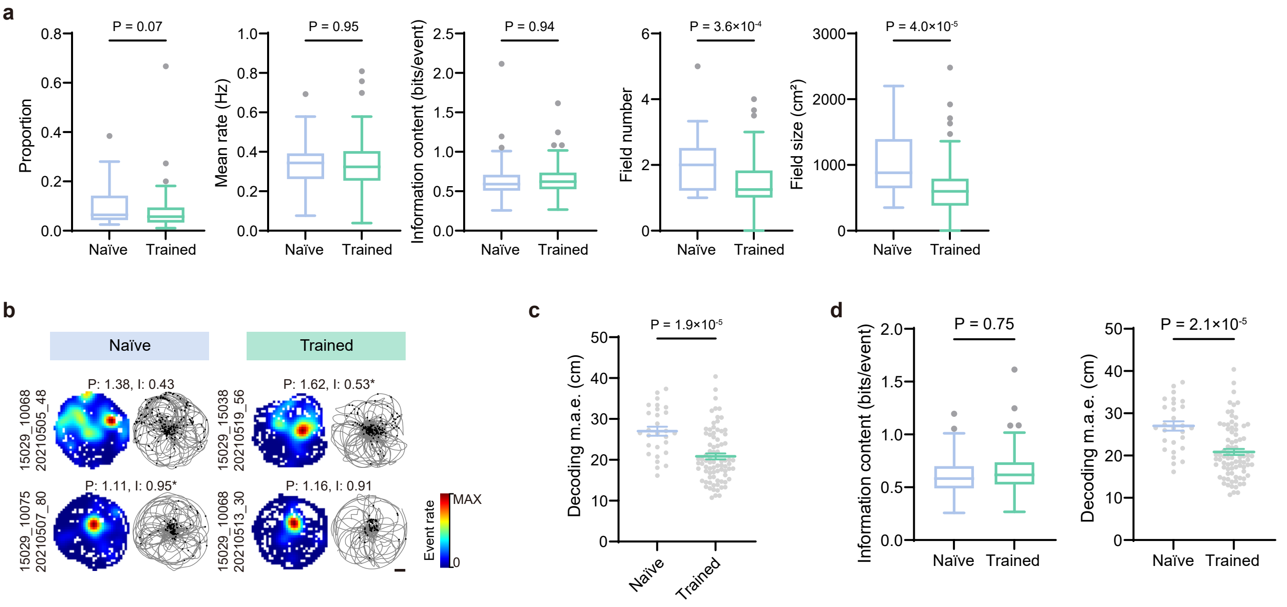

Supplementary Figure 14. Learning improves allocentric social-vector cell decoding. **a**, Comparison of the activity of alloSVCs between sessions where animals were classified as naïve (blue; 5 mice, n = 29 sessions) or trained (green; 5 mice, n = 82 sessions) (median ± i.q.r.). From left to right: fraction of cells classified as alloSVC (two-tailed Mann Whitney test, Mann-Whitney U = 919.5, Cliff's Delta = 0.23, p = 0.07), mean event rate (two-tailed Mann Whitney test, Mann-Whitney U = 1180, Cliff's Delta = 7.6×10^{-3}, p = 0.95), information content (two-tailed Mann Whitney test, Mann-Whitney U = 1178, Cliff's Delta = 9.3×10^{-3}, p = 0.94), place field number (two-tailed Mann Whitney test, Mann-Whitney U = 671.5, Cliff's Delta = 0.44, p = 3.6×10^{-4}), place field size (two-tailed Mann Whitney test, Mann-Whitney U = 593.5, Cliff's Delta = 0.50, p = 4.0×10^{-5}). **b**, Examples (one per row) of cells classified as alloSVC (asterisk) in naïve (left) and trained (right) animals. Left subpanel: event rate map. Right subpanel: trajectory (grey line) and individual events (black dots). Event rate peak (P) and information content (I) indicated. Scale bar: 20 cm. **c**, Decoding error for alloSVCs in naïve or trained mice (one dot per session, two-tailed Mann Whitney test, Mann-Whitney U = 528, Cliff's Delta = 0.53, p = 1.9×10^{-5}). **d**, as **a,c** but for alloSVCs with shuffles-in-place not classified as alloSVC (see Supplementary Fig. 6). Information content (left, two-tailed Mann Whitney test, Mann-Whitney U = 1100, Cliff's Delta = -0.04, p = 0.75) and decoding error (one dot per session, two-tailed Mann Whitney test, Mann-Whitney U = 531, Cliff's Delta = 0.53, p = 2.1×10^{-5}) for alloSVCs in naïve or trained mice. Source data are provided as a Source Data file.
