# Supplementary material for: Multiplexed representation of others in the hippocampal CA1 subfield of female mice
Source: Nat Commun. 2024 May 2;15:3702. doi: 10.1038/s41467-024-47453-8 (PMC11065873; doi:10.1038/s41467-024-47453-8)
Supplement: Supplementary file 3 — Description of additional supplementary files [file 41467_2024_47453_MOESM3_ESM.pdf]

Description of Additional Supplementary Files

Supplementary Movie 1: Behavior labeled video. The video shows DeepLabCut labeling and Boris annotation of mouse interaction in an open field. Colored dots represent the positions of behaving animals (recorded mouse: red, yellow; social partner: blue). Text in the video indicates timestamp and current behavior of the imaged mouse and its partner during social interaction.

Supplementary Movie 2: Behavior labeled video. The video shows DeepLabCut labeling and Boris annotation of mouse interaction in an open field. Colored dots represent the positions of behaving animals (recorded mouse: red, yellow; social partner: blue). Text in the video indicates timestamp and current behavior of the imaged mouse and its partner during social interaction.
