# Supplementary material for: Multiplexed representation of others in the hippocampal CA1 subfield of female mice
Source: Nat Commun. 2024 May 2;15:3702. doi: 10.1038/s41467-024-47453-8 (PMC11065873; doi:10.1038/s41467-024-47453-8)
Supplement: Supplementary file 6 — Reporting Summary [file 41467_2024_47453_MOESM6_ESM.pdf]

nature portfolio

Corresponding author(s): Chenglin Miao, Emilio Kropff

Last updated by author(s): Mar 10, 2024

Reporting Summary

Nature Portfolio wishes to improve the reproducibility of the work that we publish. This form provides structure for consistency and transparency in reporting. For further information on Nature Portfolio policies, see our Editorial Policies and the Editorial Policy Checklist.

Statistics

For all statistical analyses, confirm that the following items are present in the figure legend, table legend, main text, or Methods section.

n/a	Confirmed	
☐	☒	The exact sample size (*n*) for each experimental group/condition, given as a discrete number and unit of measurement
☐	☒	A statement on whether measurements were taken from distinct samples or whether the same sample was measured repeatedly
☐	☒	The statistical test(s) used AND whether they are one- or two-sided<br>*Only common tests should be described solely by name; describe more complex techniques in the Methods section.*
☒	☐	A description of all covariates tested
☐	☒	A description of any assumptions or corrections, such as tests of normality and adjustment for multiple comparisons
☐	☒	A full description of the statistical parameters including central tendency (e.g. means) or other basic estimates (e.g. regression coefficient) AND variation (e.g. standard deviation) or associated estimates of uncertainty (e.g. confidence intervals)
☐	☒	For null hypothesis testing, the test statistic (e.g. *F*, *t*, *r*) with confidence intervals, effect sizes, degrees of freedom and *P* value noted<br>*Give P values as exact values whenever suitable.*
☐	☒	For Bayesian analysis, information on the choice of priors and Markov chain Monte Carlo settings
☒	☐	For hierarchical and complex designs, identification of the appropriate level for tests and full reporting of outcomes
☐	☒	Estimates of effect sizes (e.g. Cohen's *d*, Pearson's *r*), indicating how they were calculated

Our web collection on statistics for biologists contains articles on many of the points above.

Software and code

Policy information about availability of computer code

Data collection	The hardware and Miniscope DAQ software of miniscope v3 (UCLA, https://github.com/Aharoni-Lab/Miniscope-DAQ-QT-Software/releases/tag/v1.10).
Data analysis	MATLAB R2020b (MathWorks), GraphPad Prism 9 (GraphPad Software), Anaconda (Continuum Analytics), Excel 2019 (Microsoft)<br>The open-source analysis pipeline to extract calcium event (https://github.com/etterguillaume/MiniscopeAnalysis, https://github.com/flatironinstitute/NoRMCorre, https://github.com/zhoupc/CNMF_E).<br>CellReg (https://github.com/zivlab/CellReg).<br>DeepLabCut (https://github.com/DeepLabCut/DeepLabCut).<br>Boris (https://github.com/olivierfriard/BORIS).<br>The Behavioural Neurology Toolbox, (c) Vadim Frolov 2018 (https://bitbucket.org/cnc-ntnu/bnt/src/master).<br>Custom written MATLAB and Python code (https://github.com/SherlockX-hub/SocialVectorCell-Public).

For manuscripts utilizing custom algorithms or software that are central to the research but not yet described in published literature, software must be made available to editors and reviewers. We strongly encourage code deposition in a community repository (e.g. GitHub). See the Nature Portfolio guidelines for submitting code & software for further information.

Data

Policy information about availability of data

All manuscripts must include a data availability statement. This statement should provide the following information, where applicable:

- Accession codes, unique identifiers, or web links for publicly available datasets
- A description of any restrictions on data availability
- For clinical datasets or third party data, please ensure that the statement adheres to our policy

Data sets supporting this paper are available in https://github.com/SherlockX-hub/SocialVectorCell-Public.

Research involving human participants, their data, or biological material

Policy information about studies with human participants or human data. See also policy information about sex, gender (identity/presentation), and sexual orientation and race, ethnicity and racism.

Reporting on sex and gender	No human data was used.
Reporting on race, ethnicity, or other socially relevant groupings	No human data was used.
Population characteristics	No human data was used.
Recruitment	No human data was used.
Ethics oversight	No human data was used.

Note that full information on the approval of the study protocol must also be provided in the manuscript.

Field-specific reporting

Please select the one below that is the best fit for your research. If you are not sure, read the appropriate sections before making your selection.

☒ Life sciences ☐ Behavioural & social sciences ☐ Ecological, evolutionary & environmental sciences

For a reference copy of the document with all sections, see nature.com/documents/nr-reporting-summary-flat.pdf

Life sciences study design

All studies must disclose on these points even when the disclosure is negative.

Sample size

For a full characterization of a novel functional cell type with unknown properties, both the number of animals and of recorded neurons (which is highly variable across animals) need to be taken into account. On top of this, the percentage that the subpopulation of interest represents over the whole population of cells is crucial. In our study, socialPCs, up to that point the only known way of representing the position of others in the hippocampus, turned out to be a very small population (around 2.3%), as a consequence of which an accurate description such as the ones included in our work required many cells. We originally aimed for around 1,500 cells in around 15 animals, based on previous novel characterizations of neural response such as that of Kropff and colleagues, 2015 (2497 cells in 17 animals) or Jercog and colleagues, 2019 (1,244 cells in 12 animals).

Kropff, E., Carmichael, J. E., Moser, M. B., & Moser, E. I. (2015). Speed cells in the medial entorhinal cortex. Nature, 523(7561), 419-424.

Jercog, P. E., Ahmadian, Y., Woodruff, C., Deb-Sen, R., Abbott, L. F., & Kandel, E. R. (2019). Heading direction with respect to a reference point modulates place-cell activity. Nature communications, 10(1), 2333.

Data exclusions

No data was excluded from the analyses.

Replication

As in all literature in this field, in our work cells from distinct animal pairs could be considered as independent but one experiment includes data from all pairs trained under the same conditions. Following standard procedures in the field we did not do replications of experiments.

Randomization

Animals were randomly allocated into each experiment group.

Blinding

The experimenters were not blinded to group allocation during data collection and analysis, but neural activity could not be observed during behavioral experiments since the raw data requires significant amounts of pre-processing. Neural activity data were obtained a posteriori using the same automatic standardized methods for animals in all conditions, reducing the possibility of bias.

Reporting for specific materials, systems and methods

We require information from authors about some types of materials, experimental systems and methods used in many studies. Here, indicate whether each material, system or method listed is relevant to your study. If you are not sure if a list item applies to your research, read the appropriate section before selecting a response.

Materials & experimental systems

n/a	Involved in the study
☐	☒ Antibodies
☒	☐ Eukaryotic cell lines
☒	☐ Palaeontology and archaeology
☐	☒ Animals and other organisms
☒	☐ Clinical data
☒	☐ Dual use research of concern
☒	☐ Plants

Methods

n/a	Involved in the study
☒	☐ ChIP-seq
☒	☐ Flow cytometry
☒	☐ MRI-based neuroimaging

Antibodies

Antibodies used	Chicken anti-GFP (1:500, Invitrogen, A10262)<br>Alexa Fluor 488 goat anti-chicken (1:500, Invitrogen, A11039)
Validation	All antibodies were validated by the manufacturer and published research articles.<br><br>Chicken anti-GFP (Invitrogen, A10262): https://www.thermofisher.cn/cn/zh/antibody/product/GFP-Antibody-Polyclonal/A10262<br>Alexa Fluor 488 goat anti-chicken (Invitrogen, A11039): https://www.thermofisher.cn/cn/zh/antibody/product/Goat-anti-Chicken-IgY-H-L-Secondary-Antibody-Polyclonal/A-11039

Animals and other research organisms

Policy information about studies involving animals; ARRIVE guidelines recommended for reporting animal research, and Sex and Gender in Research

Laboratory animals	We used C57BL/6N female mice (12 week-old; Beijing Vital River Laboratory). In order to minimize behaviors related to sex or dominance, all mice were female. All mice were group-housed with a 12h-12h light-dark cycle in a temperature controlled and air-circulating cabinet and provided with food and water as libitum. Behavioral experiments were conducted during the dark phase.
Wild animals	The study did not involve wild animals.
Reporting on sex	Gender was considered in the design of this study. Females were used to avoid confrontation between unfamiliar specimens.
Field-collected samples	The study did not involve samples collected from the field.
Ethics oversight	All procedures for animals were approved by Animal Care & Use Committees at Peking University.

Note that full information on the approval of the study protocol must also be provided in the manuscript.
